# Bioinformatics analysis of circulating miRNAs related to cancer following spinal cord injury

**DOI:** 10.1042/BSR20190989

**Published:** 2019-09-20

**Authors:** Elisangela C.P. Lopes, Layde R. Paim, José R. Matos-Souza, Décio R. Calegari, José I. Gorla, Alberto Cliquet, Carmen S.P. Lima, John F. McDonald, Wilson Nadruz, Roberto Schreiber

**Affiliations:** 1 Department of Internal Medicine, State University of Campinas Faculty of Medical Sciences, Campinas, SP, Brazil; 2 School of Physical Education, University of Maringá, Maringá, PR, Brazil; 3 School of Physical Education, State University of Campinas, Campinas, SP, Brazil; 4 Department of Orthopedics, State University of Campinas Faculty of Medical Sciences, Campinas, SP, Brazil; 5 Department of Electrical Engineering, University of São Paulo, São Carlos, SP, Brazil; 6 School of Biology, Petit Institute of Bioengineering and BioSciences, Georgia Institute of Technology, Atlanta, GA, U.S.A.

**Keywords:** cancer, miRNAs, Spinal cord injury

## Abstract

Patients with spinal cord injury (SCI) have an increased risk of developing esophageal, bladder and hematologic malignancies compared with the normal population. In the present study, we aimed to identify, through in silico analysis, miRNAs and their target genes related to the three most frequent types of cancer in individuals with SCI. In a previous study, we reported a pattern of expression of miRNAs in 17 sedentary SCI males compared with 22 healthy able-bodied males by TaqMan OpenArray. This list of miRNAs deregulated in SCI patients was uploaded to miRWALK2.0 to predict the target genes and pathways of selected miRNAs. We used Cytoscape software to construct the network displaying the miRNAs and their gene targets. Among the down-regulated miRNAs in SCI, 21, 19 and 20 miRNAs were potentially associated with hematological, bladder and esophageal cancer, respectively, and three target genes (*TP53, CCND1 and KRAS*) were common to all three types of cancer. The three up-regulated miRNAs were potentially targeted by 18, 15 and 10 genes associated with all three types of cancer. Our current bioinformatics analysis suggests the potential influence of several miRNAs on the development of cancer in SCI. In general, these data may provide novel information regarding potential molecular mechanisms involved in the development of cancer among individuals with SCI. Further studies aiming at understanding how miRNAs contribute to the development of the major cancers that affect patients after SCI may help elucidate the role of these molecules in the pathophysiology of the disease.

## Introduction

Spinal cord injury (SCI) is an insult that commonly occurs because of trauma resulting in loss or impairment of motor/sensory function below the injury level. According to the 2014 National SCI Statistical Center (NSCISC) annual report [1], neoplasms are the third leading cause of death in SCI individuals in the United States. Patients with SCI have an increased risk of developing esophageal, bladder and hematologic malignancies, such as multiple myeloma chronic or acute myeloid leukemia, and lymphoma, compared with normal population [2,3].

MicroRNAs (miRNAs) are small molecules of highly conserved non-coding RNAs, which promote post-transcriptional regulation of gene expression, and play an important role in both physiological and pathological conditions [4]. Several studies have demonstrated the expression of aberrant miRNA in most human malignancies, so that some highly expressed miRNAs can function as oncogenes by suppressing tumor suppressor genes [5]. By contrast, some miRNAs, even when expressed at low levels, can function as tumor suppressors by down-regulating oncogenes [5]. However, the miRNAs and their target genes in oncogenic pathways involved in the development of neoplasms among SCI individuals have not been clearly elucidated. Knowledge regarding the molecular mechanisms of initiation and progression of cancers in patients with SCI can provide potential information on therapeutic approaches and biomarkers for the preventive diagnosis of SCI-related cancer.

In a previous study, we reported for the first time a specific pattern of circulating miRNAs expression in individuals with chronic SCI compared with healthy controls [6]. Given that patients with SCI have higher risk of cancer and miRNAs have been implicated in the risk of cancer, it is possible that the distinct pattern of miRNA expression in SCI might be involved in the development of cancer in this population. Therefore, in the present study, we performed an exploratory analysis aiming to identify miRNAs and their target genes related to the three most frequent types of cancer in SCI individuals, using bioinformatics tools, among the miRNAs that were reported to be differently expressed in individuals with SCI compared with healthy individuals [6].

## Materials and methods

### MiRNA expression microarray data

We previously evaluated 17 sedentary SCI (SCI-S) males with 1 year or more of SCI and 22 apparently healthy able-bodied males [6]. Twenty-six deregulated (23 down-regulated and 3 up-regulated) circulating miRNAs were identified in SCI individuals when compared with healthy controls using a TaqMan OpenArray® Human MicroRNA system (Life Technologies). These miRNAs, which are also presented in Supplementary Table S1, were used for the current analysis. Detailed clinical characteristics of the patients used for the analysis of miRNAs can be found elsewhere [6].

### Identification of miRNA target genes

Potential target genes regulated by the differentially expressed miRNAs between SCI-S and healthy individuals were predicted using the miRWalk 2.0 online tool [7]. To understand the biological relevance of differentially expressed miRNAs, we performed functional enrichment analysis for each of the three most common types of cancer in patients with SCI (bladder, esophageal and hematologic cancer). To strengthen the data, only mRNAs predicted in at least four out of five tools (miRanda, miRDB, miRWalk, RNA22 and TargetScan) were considered as possible miRNA targets. In this way, we obtained the target genes for specific miRNAs. Additionally, a network displaying the miRNAs and their gene targets was constructed and visualized using Cytoscape software [8].

## Results

### Prediction of miRNA target genes

**Figure 1:**
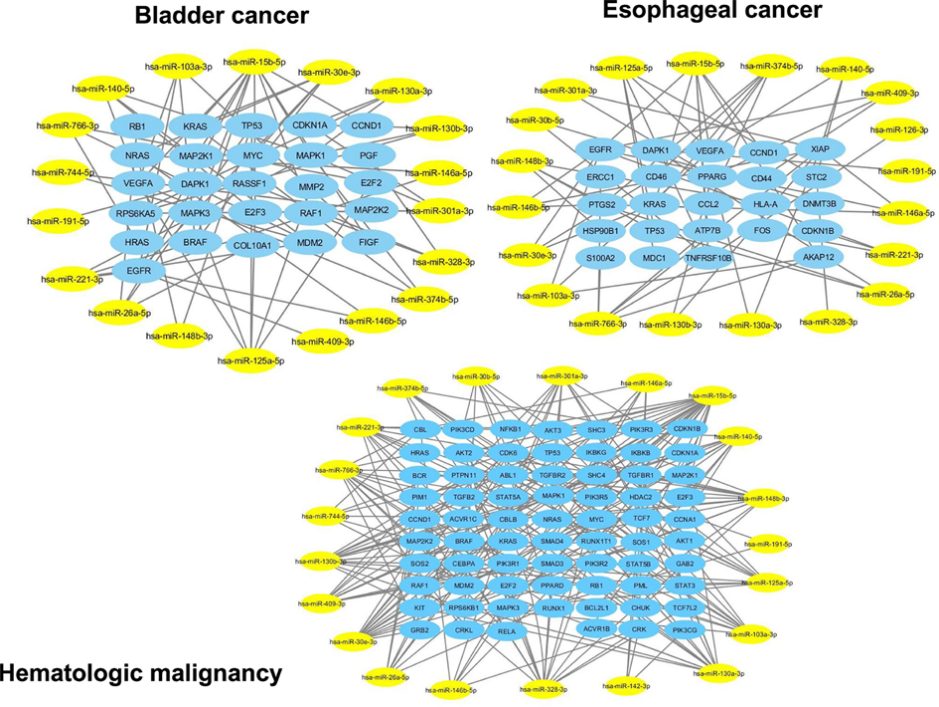
Regulatory network of down-regulated miRNAs in SCI individuals and target genes associated with cancer (bladder, esophageal and hematologic malignancies) Yellow ellipse nodes represent differentially expressed miRNAs. Blue ellipse nodes represent target genes.

**Figure 2:**
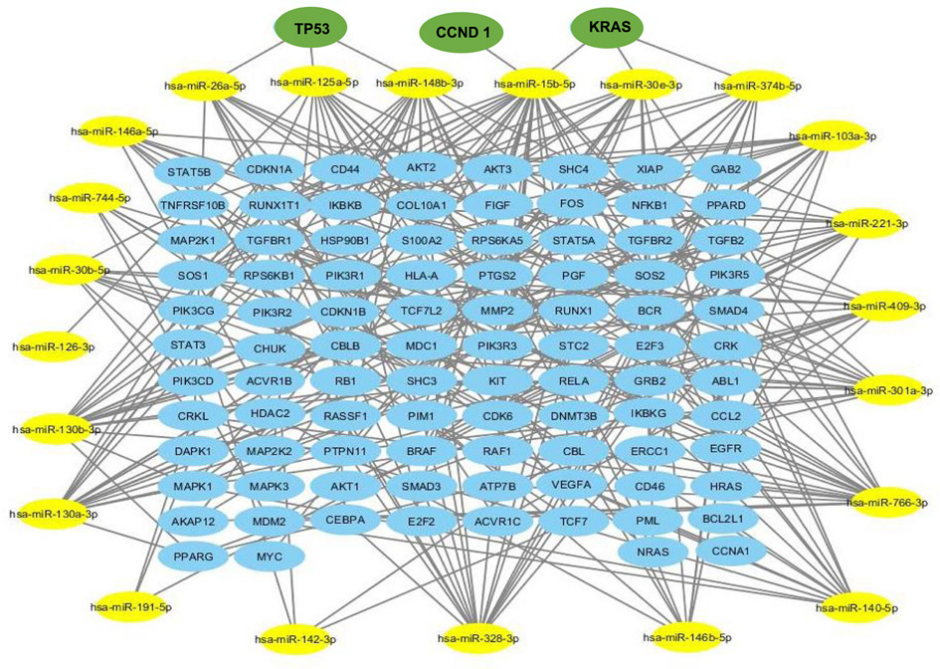
Combination of regulatory network of down-regulated miRNAs in SCI individuals and target genes associated with all studied cancers (bladder, esophageal and hematologic malignancies) Yellow ellipse nodes represent differentially expressed miRNAs. Blue ellipse nodes represent target genes. The green nodes represent the target genes common to the three types of cancer.

Using the miRWalk, miRanda, miRDB, RNA22 and TargetScan databases, of the 23 miRNAs down-regulated in SCI-S, only one was not associated with one of the three types of cancer studied and 21 were associated with hematologic cancer, 19 with bladder cancer and 20 with esophageal cancer. These miRNAs regulated 69, 26 and 24 target genes, respectively (Figure 1). The combination of all target genes demonstrated that the *TP53, CCND1 and KRAS* genes were common to all three types of cancer (Figure 2).

**Figure 3:**
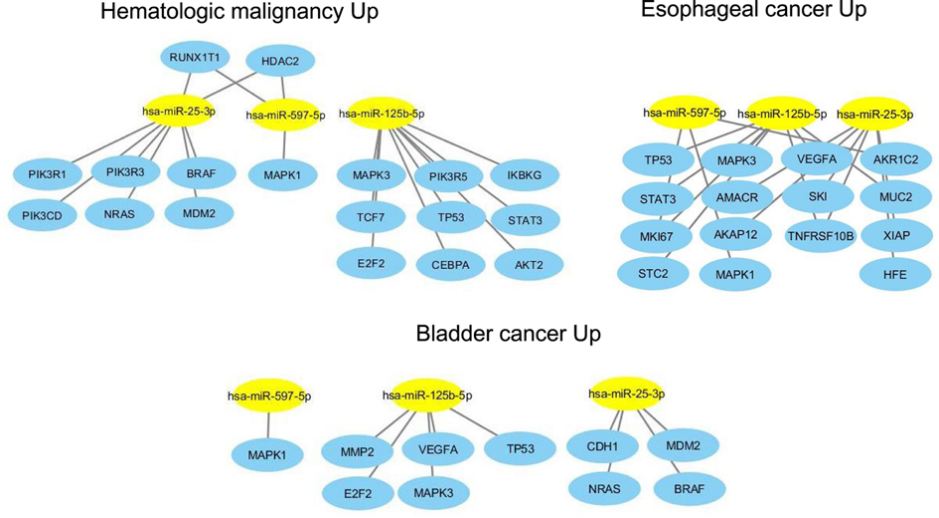
Regulatory network of up-regulated miRNAs in SCI individuals and targets genes associated with cancer (bladder, esophageal and hematologic malignancies) Yellow ellipse nodes represent differentially expressed miRNAs. Blue ellipse nodes represent targets genes.

**Figure 4:**
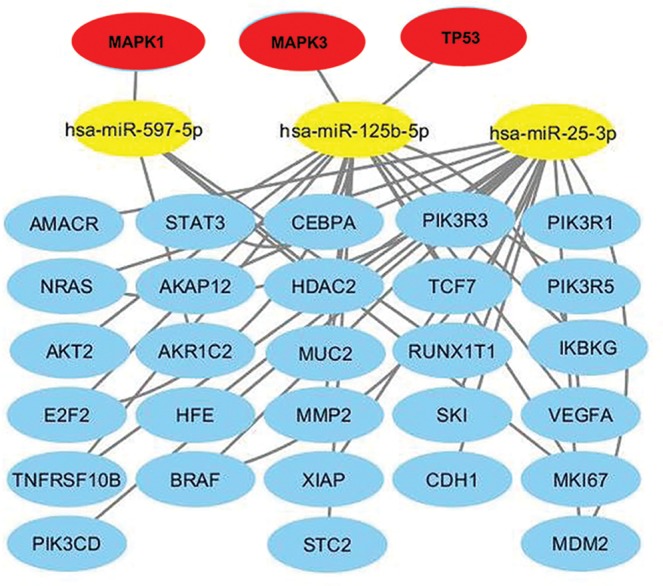
Combination of regulatory network of up-regulated miRNAs in SCI individuals and targets genes associated with all of cancer (bladder, esophageal and hematologic malignancies) Yellow ellipse nodes represent differentially expressed miRNAs. Blue ellipse nodes represent targets genes. The red nodes represent the target genes common to the three types of cancer.

Analysis of miRNAs up-regulated in SCI individuals (miR-125b-5p; miR-597-5p; miR-25-3p) resulted in 18 target genes associated with hematologic cancer, 10 with bladder cancer and 15 with esophageal cancer (Figure 3). The combination of all target genes demonstrated that the *TP53, MAPK1 and MAPK3* genes were common to all three types of cancer (Figure 4).

## Discussion

In the present study, a bioinformatics analysis was performed to identify potential miRNAs and their target genes related to the three most frequent types of cancer in individuals with SCI. For this purpose, we analyzed the differentially deregulated circulating miRNAs in individuals with SCI from a previous study of our group [6] and showed that among the down-regulated miRNAs in SCI, 21, 19 and 20 miRNAs were potentially associated with hematological, bladder and esophageal cancer, respectively.

Increased risk of bladder cancer has been reported, ranging from 16 to 28 times in patients with SCI compared with the able-bodied population, even in patients without neurogenic bladder [3,9]. Several studies have demonstrated down-regulation of miR-26a, miR-15b-5p and miR-374b-5p associated with the development of bladder cancer in general populations [10–15]. These miRNAs were down-regulated in our studied SCI individuals suggesting that they may be potential regulators of genes related to bladder cancer in these patients. In addition, these miRNAs have been associated with regulation of *TP53* [16], *CCND1* [13] and *VEGFA* [17] in bladder cancer supporting our *in silico* analysis showed that these genes might be potential targets of down-regulated miRNAs in SCI individuals.

A greater risk of hematologic and esophageal cancer has been reported in patients with SCI [2], but the mechanisms underlying these associations are not established. A former study has shown that miR-125a-5p have a reduced expression in patients with esophageal squamous cell carcinoma [18] suggesting that this miRNA can function as a tumor suppressor in this type of cancer. In our study, this miRNA was reduced in serum of SCI individuals and *in-silico* analysis suggested five potential gene targets regulated by this miRNA (*TP53, MDC1, STC2, TNFRSF10B e VEGFA*). Furthermore, miR-25, an up-regulated miRNA in our sample of SCI individuals, may promote migration and invasion of esophageal squamous cell carcinoma cells by suppressing the expression of the *CDH1* gene [19]. Consistent with this notion, our results of *in silico* analysis pointed toward *CDH1* as a potential target for this miRNA.

In our *in silico* analysis, we observed 21 down-regulated miRNAs in SCI individuals that may regulate the expression of 69 potential gene targets associated with hematologic cancer, including acute myeloid leukemia and chronic myeloid leukemia. The miR-15/16 cluster, miR-146a, miR-328 and miR-125b have been associated with development of hematologic cancer [20–26]. In the present study, mir-15b-5p, miR-146a-5p and miR-328 were down-regulated in SCI individuals and our *in silico* analysis identified several potential targets genes regulated for these miRNAs, including the oncogene *PIM1* [24], while miR-125b was up-regulated in SCI individuals and *in silico* analysis revealed nine target genes as potential targets of this miRNA associated with hematological disease, including tumor suppressor genes such as *TP53, STAT3 and TCF7* [27].

Notably, three target genes (*TP53, CCND1 and KRAS*) were common to all three types of cancer. When considering the up-regulated miRNAs in SCI patients, three distinct target genes (*MAPK1, MAPK3 and TP53*) were common to all three types of cancer. Our *in silico* analysis showed that down-regulation of miR-15b-5p, miR-30e-3p and miR-374b-5p in SCI individuals may have *KRAS* as one of the targets gene presents in the three most frequent types of cancer related to SCI. Thus, we speculate that the *KRAS gene*, may be an attractive target for further studies evaluating oncogenesis in SCI individuals.

The difficulty in obtaining tumor samples in patients who have developed cancer after SCI in addition to the fact that circulating miRNAs do not always originate from tumor tissue [28] are limitations of our study, since our miRNA analysis was performed in the serum of patients with SCI who did not have a diagnosis of cancer. Another limitation relates to the fact that *in silico* analysis alone is not enough to elucidate the role of these molecules in the pathophysiology of cancer in SCI, suggesting that further *in vitro* and *in vivo* studies should be performed to confirm the role of these miRNAs in the development of cancer associated with SCI.

To date, we are unaware of any study that has evaluated the association of miRNA expression pattern with any type of cancer in SCI patients. The aim of our study was to investigate whether miRNAs that have been already related to cancer development in the general population might be also potentially related to cancer in SCI individuals. Since our study is a hypothesis-generating research, these findings might help to elucidate the role of these miRNAs in the increased prevalence of cancer in patients with SCI.

Understanding the regulatory roles of miRNAs has already been demonstrated in several types of cancer, however few studies have demonstrated the influence of miRNAs on the development of cancer in SCI individuals to date. Our current bioinformatics analysis suggests the potential influence of several of miRNAs on the development of cancer in individuals with SCI. Further studies aiming at understanding how miRNAs contribute to the development of the major cancers that affect patients after SCI may help elucidate the role of these molecules in the pathophysiology of the disease.

## Supporting information

**Supplementary Table S1:** Differentially expressed miRNAs between sedentary individuals with spinal cord injury (SCI-S) and able-bodied individuals (AB) and their associations with cancer types.

## Abbreviations

miRNA: microRNA
NSCISC: National SCI Statistical Center
SCI: spinal cord injury
SCI-S: sedentary spinal cord injury
