# Supplementary material for: Bioinformatics analysis of circulating miRNAs related to cancer following spinal cord injury
Source: Biosci Rep. 2019 Sep 20;39(9):BSR20190989. doi: 10.1042/BSR20190989 (PMC6753324; doi:10.1042/BSR20190989)
Supplement: Supplementary file 1 [file bsr20190989_Supp1.pdf]

Bioinformatic analysis of circulating miRNAs related to cancer following spinal cord injury

Elisangela C.L. Dias, MSc[1]; Layde R. Paim, PhD[1]; José R. Matos-Souza, PhD[1]; Décio R. Calegari, PhD[2]; José I. Gorla, PhD[3]; Alberto Cliquet Jr., PhD[4,5]; Carmen S.P. Lima PhD[1], John F. McDonald PhD[6], Wilson Nadruz Jr., PhD[1]; Roberto Schreiber, PhD[1]

Supplemental Material

Supplementary Table 1. Differentially expressed miRNAs between sedentary individuals with spinal cord injury (SCI-S) and able-bodied individuals (AB) and their associations with cancer types.

	SCI-S X AB			
	Specific to Bladder Cancer	Specific to esophageal Cancer	Specific to hematologic Cancer	All Cancers
	Downregulated miRNAs			
miR-766-3p	X	X	X	X
miR-409-3p	X	X	X	X
miR-374b-5p	X	X	X	X
miR-328-3p	X	X	X	X
miR-30e-3p	X	X	X	X
miR-301a-3p	X	X	X	X
miR-26a-5p	X	X	X	X
miR-221-3p	X	X	X	X
miR-191-5p	X	X	X	X
miR-15b-5p	X	X	X	X
miR-148b-3p	X	X	X	X
miR-146b-5p	X	X	X	X
miR-146a-5p	X	X	X	X
miR-140-5p	X	X	X	X
miR-130b-3p	X	X	X	X
miR-130a-3p	X	X	X	X
miR-125a-5p	X	X	X	X
miR-103a-3p	X	X	X	X
miR-744-5p	X		X	
miR-30b-5p		X	X	
miR-142-3p			X	
miR-126-3p		X		
miR-127-3p				
	Upregulated miRNAs			
miR-597-5p	X	X	X	X
miR-125b-5p	X	X	X	X
miR-25-3p	X	X	X	X

Legend: Differentially expressed miRNAs between SCI-S and AB individuals according to reference 6 (Paim LR et al. J Neurotrauma 2018, 35:1–8) and their associations with different cancer types as assessed by miRWalk 2.0.
